# A comparison of estimators from self-controlled case series, case-crossover design, and sequence symmetry analysis for pharmacoepidemiological studies

**DOI:** 10.1186/s12874-017-0457-7

**Published:** 2018-01-08

**Authors:** Yoshinori Takeuchi, Tomohiro Shinozaki, Yutaka Matsuyama

**Affiliations:** 1 Department of Biostatistics, School of Public Health, Graduate School of Medicine, The University of Tokyo, 7-3-1, Hongo, Bunkyo-ku, Tokyo, Japan; 2 Department of Healthcare Information Management, The University of Tokyo Hospital, 7-3-1, Hongo, Bunkyo-ku, Tokyo, 113-8655 Japan

**Keywords:** Bias, Interaction, Medical database, Misspecified risk period, Restriction, Self-controlled methods, Time-invariant confounding, Time trends, Time-varying confounding

## Abstract

**Background:**

Despite the frequent use of self-controlled methods in pharmacoepidemiological studies, the factors that may bias the estimates from these methods have not been adequately compared in real-world settings. Here, we comparatively examined the impact of a time-varying confounder and its interactions with time-invariant confounders, time trends in exposures and events, restrictions, and misspecification of risk period durations on the estimators from three self-controlled methods. This study analyzed self-controlled case series (SCCS), case-crossover (CCO) design, and sequence symmetry analysis (SSA) using simulated and actual electronic medical records datasets.

**Methods:**

We evaluated the performance of the three self-controlled methods in simulated cohorts for the following scenarios: 1) time-invariant confounding with interactions between the confounders, 2) time-invariant and time-varying confounding without interactions, 3) time-invariant and time-varying confounding with interactions among the confounders, 4) time trends in exposures and events, 5) restricted follow-up time based on event occurrence, and 6) patient restriction based on event history. The sensitivity of the estimators to misspecified risk period durations was also evaluated. As a case study, we applied these methods to evaluate the risk of macrolides on liver injury using electronic medical records.

**Results:**

In the simulation analysis, time-varying confounding produced bias in the SCCS and CCO design estimates, which aggravated in the presence of interactions between the time-invariant and time-varying confounders. The SCCS estimates were biased by time trends in both exposures and events. Erroneously short risk periods introduced bias to the CCO design estimate, whereas erroneously long risk periods introduced bias to the estimates of all three methods. Restricting the follow-up time led to severe bias in the SSA estimates. The SCCS estimates were sensitive to patient restriction. The case study showed that although macrolide use was significantly associated with increased liver injury occurrence in all methods, the value of the estimates varied.

**Conclusions:**

The estimations of the three self-controlled methods depended on various underlying assumptions, and the violation of these assumptions may cause non-negligible bias in the resulting estimates. Pharmacoepidemiologists should select the appropriate self-controlled method based on how well the relevant key assumptions are satisfied with respect to the available data.

**Electronic supplementary material:**

The online version of this article (10.1186/s12874-017-0457-7) contains supplementary material, which is available to authorized users.

## Background

Medical databases, including medical claims data and electronic medical records, are increasingly used in pharmacoepidemiological research to assess the associations between drug exposure and adverse effects. The clear advantage of these database studies is the provision of large sample sizes enabling the evaluations of rare adverse events. On the other hand, the associations between specific drugs and adverse events may be confounded by time-invariant factors, including genetic factors, chronic medical conditions, and patient lifestyle. Because medical databases rarely contain such information, it is difficult to adjust for these time-invariant confounders.

Self-controlled methods, which are also referred to as case-only methods, are observational study designs where the case patients who experience the event of interest act as their own controls based on data from non-case periods [1–3]. The use of cases as their own controls minimizes the confounding effects of time-invariant risk factors. Self-controlled methods may therefore represent a powerful option for analyzing routinely collected medical databases that lack information on potentially influential confounders [1].

**Fig. 1:**
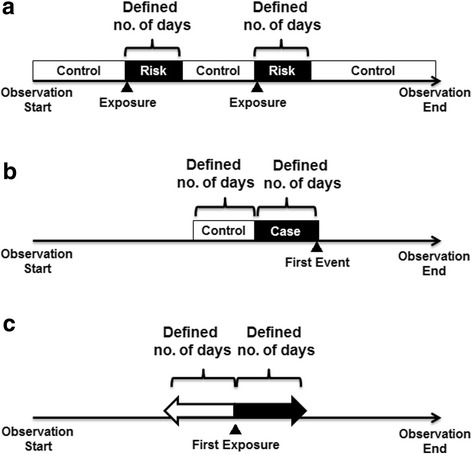
Typical settings of analytical periods for the three self-controlled methods used in this study. **a** Self-controlled case series. The observation period of each subject is divided into risk periods (defined number of days after an exposure) and control periods (all other periods). **b** Case-crossover design. For each case, a case period (defined number of days before the first event) and a corresponding control period (defined number of days before the first event) are designated. (c) Sequence symmetry analysis. A crude sequence ratio is calculated by dividing the number of subjects who had experienced the first event a defined number of days after the first exposure (indicated by the black arrow, i.e., exposure ➔ event) by the number of subjects who had experienced the first event a defined number of days before the first exposure (indicated by the white arrow, i.e., event ➔ exposure)

Among the existing self-controlled methods, self-controlled case series (SCCS) [4, 5], case-crossover (CCO) design [6, 7], and sequence symmetry analysis (SSA) [8, 9] are frequently employed in pharmacoepidemiological studies. Although all three methods estimate the relative risk or odds ratio of an event during exposure periods compared with non-exposure periods, they differ in their designated analytical periods. In SCCS, the relative risk during the exposure (risk) and non-exposure (control) periods are estimated. In CCO design, the odds of exposure in the time period immediately preceding the event of interest (case period) are compared with those in an earlier time period that did not result in an event (control period). In SSA, a crude sequence ratio is calculated as an effect measure of relative risk by dividing the number of subjects who had experienced the first event after the first exposure by the number of subjects who had experienced the first event before the first exposure. The typical designs of these methods are illustrated in Fig. 1.

In pharmacoepidemiological studies, the estimates of the three self-controlled methods may be biased under the following situations: the presence of short-term time-varying factors, such as the transient use of concomitant drugs or acute disease occurrence; the influence of long-term factors, such as seasonality, age effects, and time trends in drug prescriptions; restricted follow-up of patients based on event occurrence; patient restriction based on event history; and the misspecification of risk period durations (i.e., erroneously short or long). Although previous review articles have described several assumptions for appropriate estimations using these methods [1, 2, 10], few studies have statistically compared the performance of the methods using a single data source for the aforementioned situations.

In this study, we simulated several databases containing electronic medical records to compare the estimators produced by SCCS, CCO design, and SSA in the presence of a short-term time-varying confounder and its interactions with time-invariant confounders, as well as in the presence of long-term time trends in exposures and events. Additionally, we examined the effects of sequentially censoring patients at the time of each event and restricting analyses to patients who did not experience the event before the first exposure. Moreover, the sensitivity of the estimators to misspecified risk period durations of exposure was evaluated. Finally, these three methods were also compared using real-world data extracted from a university hospital’s electronic medical records database for estimating the association between macrolide use and liver injury.

## Methods

### Self-controlled methods

Table 1 provides an overview of the three self-controlled methods used in this study. The SCCS method identifies patients who experienced an event at least once during the designated study duration, and models a Poisson distribution during the period *k* of patient *i* with expected events. This is described in Eq. (1) as follows:

1 $$ {\lambda}_{ik}={t}_{ik}\exp \left\{{\phi}_i+\beta {X}_i(k)\right\} $$

where *t*_*ik*_ represents the length of period *k* (as an offset term), exp(*ϕ*_*i*_) the baseline (i.e., unexposed) incidence rate, and *X*_*i*_(*k*) the exposure status of period *k* (0 for the control period and 1 for the exposure period) of patient *i* [11]. In the calculation of the likelihood function conditional on becoming a case [11], the individual patient effect *ϕ*_*i*_ is cancelled out because each patient acts as their own control; therefore, the incident rate ratio (IRR) *β* for exposure *X*_*i*_(*k*) can be estimated without estimation of *ϕ*_*i*_. A previous simulation study showed that the SCCS method was less sensitive to time trends in probability for exposures and/or events, even for small sample sizes [12]. However, the effect estimates can become biased if a prior event affects subsequent exposure probability, event rate, or both [13, 14].

**Table 1:** Overview of the three self-controlled methods

Methods	Effect measures	Study populations	Information used in analysis^a^	Main assumptions
Self-controlled case series	Incident rate ratio	Case patients who experienced at least one event during each observation period	Every exposure and every event for each case patient	Events do not alter the probability of subsequent exposure and events.
Case-crossover design	(Exposure) odds ratio	Case patients who experienced at least one event during each observation period	Every exposure and the first event for each case patient	There are no time-trends in the occurrence of exposure.
Sequence symmetry analysis	Adjusted sequence ratio	Case patients who experienced at least one exposure or one event during each observation period	The first exposure and first event for the study population	Events do not alter the probability of subsequent exposure. Trends in the occurrence of exposure and events are similar to those for the study population.

^a^Minimal information required for the calculation of each effect measure

In CCO design, a patient’s exposure experience during the case period is compared with his/her exposure experience during the control period, and the exposure probability *π*_*ij*_ in the period *j*_*i*_ (1 if case period, 0 if control period) of patient *i* is modelled as follows in Eq. (2):

2 $$ \mathrm{logit}\left({\pi}_{ij}\right)={\varphi}_i+\gamma \times {j}_i $$

where expit(*φ*_*i*_) represents the patient *i*-specific exposure probability during control periods [15]. Analogous to matched case-control studies, the conditional exposure odds ratio (OR) of case and control periods *γ* coincides with the conditional event OR between exposed and unexposed periods. As with the SCCS, the individual patient effect *φ*_*i*_ is cancelled out in the conditional likelihood function. Note that asymptotically unbiased estimations require assumptions that the exposure has no carryover effects and that the confounders are independent to exposure trends [15, 16]. Moreover, bias can be introduced to the CCO design estimates if the exposure prevalence is different between the case and control periods, i.e., if an exposure time trend is present [16, 17].

The SSA method was developed to examine the symmetry in the distribution of an event before and after an exposure of interest [18]. That initial report argued that the ratio of exposure-event sequence orders approximates the IRR in exposed and non-exposed person-time without algebraic proof [18]. The use of source population trends in exposures or events is recommended to adjust for time trends in the occurrence of these episodes [19], which may alter SSA estimates [18, 20]. Although SSA has been frequently used in pharmacoepidemiological studies [1, 8, 9], the performance of this method as an IRR estimator in the pharmacoepidemiological context remains unclear. The estimation methods and underlying models are described in the “Analysis of simulated datasets” subsection.

### Simulation study design

We used a simulation study setting designed to evaluate the risk of an acute event *Y*(*t*) (e.g., liver injury, or LI) associated with a transient exposure *X*(*t*) (e.g., antibiotic use) at day *t* = 1, …, 1800. Two time-invariant binary confounders *C*_1_ and *C*_2_ (e.g., genetic factors, chronic conditions, or sex) and one binary time-varying confounder *C*_3_(*t*) (e.g., brief administration of drugs or occurrence of an acute disease) were also generated.

**Fig. 2:**
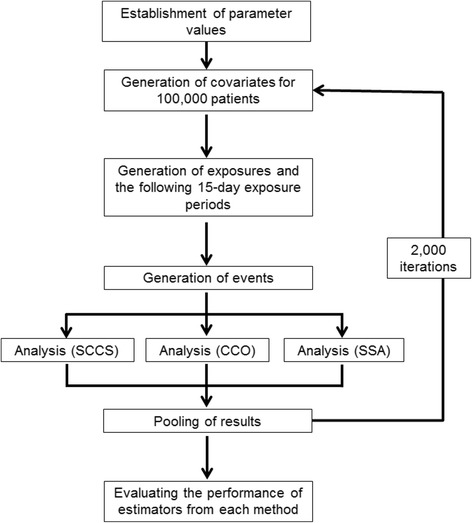
Summary of the data generation and analytical process used in the simulation study. Abbreviations: CCO, case-crossover; SCCS, self-controlled case series; SSA, sequence symmetry analysis

For each simulation setting, we simulated 2000 datasets, each of which was composed of 100,000 patients who had an 1800-day observation period without drop-outs or censoring (Fig. 2). First, *C*_1_ and *C*_2_ were generated at Day 0 using a Bernoulli distribution with success probabilities of 0.05 and 0.5, respectively; their values were kept constant throughout the observation period. Time to onset of *C*_3_(*t*) followed an exponential distribution with a rate of 0.001, and was re-generated immediately after *C*_3_(*t*) = 1 for subjects who had already experienced an onset of *C*_3_(*t*) (this allows for more than one onset of *C*_3_(*t*) for each subject). We assumed that *C*_3_(*t*) occurred over a short period of time. Therefore, we set a fixed condition of “*C*_3_(*t*) = 1” during a defined duration (covariate-effect time; *Et*_*C*_) that began from the onset of *C*_3_(*t*). If a new *C*_3_*(t)* was initiated before the fixed period of the previous *C*_3_*(t)* had ended, that fixed period was extended by the defined duration (*Et*_*C*_).

The incidence of exposure *X*(*t*) was generated using a piecewise exponential distribution with rate parameters, as described in Eq. (3).

3 $$ {\lambda}_X(t)=\exp \left\{\log \left({2.5}^{\ast }{10}^{-4}\right)+\log (2.0){C}_1+\log (1.2){C}_2+\log (5.0){C}_3(t)\right\} $$

The exposure can occur multiple times; once an exposure began, the next time-to-exposure was generated using the above exponential distribution. For this study, we assumed that the exposure *X*(*t*) is the usage of antibiotics that would have a transient effect on the incidence of an LI event. A fixed condition of “*X*(*t*) = 1” was set during the defined duration (exposure-effect time; *Et*_X_ = 15 days) that began from the onset of *X*(*t*). If a new exposure occurred before the fixed period of the previous *X*(*t*) had ended, that fixed period was extended by the defined duration (*Et*_*X*_).

Finally, event *Y*(*t*) followed a piecewise exponential distribution with rate parameters as described in Eq. (4).

4 $$ {\displaystyle \begin{array}{r}{\lambda}_Y(t)=\exp \Big\{\log \left({2.0}^{\ast }{10}^{-5}\right)+{\beta}_XX(t)+{\beta}_{C1}{C}_1+{\beta}_{C2}{C}_2+{\beta}_{C3}{C}_3(t)+{\beta}_{C1C2}{C}_1{C}_2\\ {}+{\beta}_{C2C3}{C}_2{C}_3(t)\left)\right\}\end{array}} $$

where the parameters varied according to the scenarios described below. Events can occur multiple times; once an event occurred, the next time-to-event was generated using the above exponential distribution. Note that for the data generation step in this study, the assumption holds that the occurrence of an event did not affect the probability of subsequent exposure, which is considered a key assumption for SCCS and SSA.

### Simulation scenarios for time-varying confounding

The following scenarios were utilized to evaluate the effects of a time-varying confounder on the estimates from the three methods.

#### Scenario 1

There is time-invariant confounding (*β*_*C*1_ = log(2.0), *β*_*C*2_ = log(3.0)) and interactions between the time-invariant confounders (*β*_*C*1C2_ = log(5.0)); the effect of exposure *β*_*X*_ is varied (log(1.0), log(3.0) or log(10.0)).

#### Scenario 2

There is time-invariant (*β*_*C*1_ = log(2.0), *β*_*C*2_ = log(3.0)) and time-varying confounding but no interactions; the effect of exposure *β*_*X*_ (log(1.0), log(3.0) or log(10.0)), the effect of time-varying confounder *β*_*C*3_ (log(0.2), log(0.5), log(2.0) or log(5.0))_,_ and the effect of covariate-effect time *Et*_C_ (5, 10, 15, 20 or 30 days) are varied. When changing the parameters *β*_*X*_, *β*_*C*3_, or *Et*_C_, the other parameters are fixed.

#### Scenario 3

In addition to time-invariant and time-varying confounding (*β*_*C*1_ = log(2.0), *β*_*C*2_ = log(3.0), *β*_*C*3_ = log(5.0)), there are interactions among the time-invariant and time-varying confounders; the effect of *β*_*X*_ (log(1.0), log(3.0) or log(10.0)), the interaction effects among the time-invariant and time-varying confounders *β*_*C*2*C*3_ (log(0.2), log(0.5), log(2.0) or log(5.0))_,_ and the effect of covariate-effect time *Et*_C_ (5, 10, 15, 20 or 30 days) are varied. When changing the parameters *β*_*X*_, *β*_*C*2*C*3_, or *Et*_C_, the other parameters are fixed.

##### Simulation scenario for long-term time trends in exposures and events

For the evaluation of time trends in exposures and events, we slightly altered the data generation step. Specifically, the incidence of exposure *X*(*t*) was generated using Eq. (5).

5 $$ {\lambda}_X(t)=\exp \left\{\log \left({2.5}^{\ast }{10}^{-4}\right)+\log (2.0){C}_1+\log (1.2){C}_2+\log (5.0){C}_3(t)+{\alpha}_{\mathrm{TR}}t\right\} $$

where *α*_TR_ is the effect of time trend on the onset of exposure. Event *Y*(*t*) was subsequently generated using Eq. (6).

6 $$ {\lambda}_Y(t)=\exp \left\{\log \left({2.0}^{\ast }{10}^{-5}\right)+{\beta}_XX(t)+{\beta}_{C1}{C}_1+{\beta}_{C2}{C}_2+{\beta}_{C3}{C}_3(t)+{\beta}_{C1C2}{C}_1{C}_2+{\beta}_{C2C3}{C}_2{C}_3(t)+{\beta}_{\mathrm{TR}}t\right\} $$

where *β*_TR_ is the effect of time trend on event occurrence. Based on this data generation process, we established the following scenario:

#### Scenario 4

There are time-invariant confounding (*β*_*C*1_ = log(2.0), *β*_*C*2_ = log(3.0)) and time trends in exposures or events; the time trend of exposure *α*_TR_ or events *β*_TR_ (0 or log(1.001)) are varied. Table 2 summarizes the detailed parameters of Scenarios 1 to 4 in the simulation study.

**Table 2:** Detailed Parameters of Scenarios 1 to 4 in the Simulation Study

Scenarios	*β*_*X*_	*β*_*C*1_	*β*_*C*2_	*β*_*C*3_	*β*_*C*1*C*2_	*β*_*C*2*C*3_	*α*_TR_	*β*_TR_	*Et*_*C*_ (Days)^a^
1	log(1.0)	log(2.0)	log(3.0)	log(1.0)	log(5.0)	log(1.0)	–	–	15
	log(3.0)						–	–	
	log(10.0)						–	–	
2	log(1.0)	log(2.0)	log(3.0)	log(5.0)	log(1.0)	log(1.0)	–	–	15
	log(3.0)						–	–	
	log(10.0)						–	–	
	log(3.0)	log(2.0)	log(3.0)	log(0.2)	log(1.0)	log(1.0)	–	–	15
				log(0.5)			–	–	
				log(2.0)			–	–	
	log(3.0)	log(2.0)	log(3.0)	log(5.0)	log(1.0)	log(1.0)	–	–	5
							–	–	10
							–	–	20
							–	–	30
3	log(1.0)	log(2.0)	log(3.0)	log(5.0)	log(1.0)	log(5.0)	–	–	15
	log(3.0)						–	–	
	log(10.0)						–	–	
	log(3.0)	log(2.0)	log(3.0)	log(5.0)	log(1.0)	log(0.2)	–	–	15
						log(0.5)	–	–	
						log(2.0)	–	–	
	log(3.0)	log(2.0)	log(3.0)	log(5.0)	log(1.0)	log(5.0)	–	–	5
							–	–	10
							–	–	20
							–	–	30
4	log(3.0)	log(2.0)	log(3.0)	log(1.0)	log(1.0)	log(1.0)	log(1.001)	log(1.0)	15
							log(1.0)	log(1.001)	
							log(1.001)	log(1.001)	

^a^Length of the period in which the time-varying covariate *C*_3_*(t)* has an effect

##### Simulation scenario for restricting follow-up time based on event occurrence

In Scenarios 1 to 4, all patients had the same observation period (1800 days). However, this setting is not applicable if the observations can be censored due to patient death associated with the event of interest (e.g., myocardial infarction). The restriction of follow-up time based on event occurrence may affect the estimators of SCCS and SSA. To evaluate the effect of this condition, we probabilistically censored the observation of patients when an event occurred. Based on this data generation process, we established the following scenario:

#### Scenario 5

There is time-invariant confounding (*β*_*C*1_ = log(2.0), *β*_*C*2_ = log(3.0)) but no time trends (*α*_TR_ = *β*_TR_ = 0); the observation of patients was sequentially censored upon event occurrence. Censoring probability (*P*_*C*_) was based on the Bernoulli distribution with success probabilities of 0.3, 0.6 or 1.0.

Note that a *P*_*C*_ value of 1.0 would mean that all subjects were censored at their first event (e.g., the event of interest is death). Therefore, SSA could not be performed when *P*_*C*_ was set to 1.0 because no patients would experience this event before their first exposure under this setting.

##### Simulation scenario for patient restriction based on event history

In pharmacoepidemiological studies, analyses may be restricted to subjects who did not experience the event before their first exposure if the analyst suspects that the event alters the probabilities of subsequent exposures or events. To compare the estimates of self-controlled methods under this restriction, the following scenario was utilized:

#### Scenario 6

There is time-invariant confounding (*β*_*C*1_ = log(2.0), *β*_*C*2_ = log(3.0)) but no time trends (*α*_TR_ = *β*_TR_ = 0); patients who experienced the first event before their first exposure were excluded from the study population.

This scenario could not be applied to SSA because the crude sequence ratio (CSR) cannot be calculated if the number of patients who had experienced the first event before their first exposure is zero.

### Analysis of simulated datasets

For the SCCS, risk periods were defined as a 15-day period that began on the first day of an exposure, and the control period encompassed all other periods outside of the risk periods. The IRR for exposure *β*_*X*_ and 95% confidence intervals (CI) were estimated using a univariable conditional Poisson regression model stratified by each case, and the risk periods and control periods were compared [11]. In this analysis, we used SAS macro programs (element.sas, sccs.sas, and poisreg.sas) available from the Open University website, UK [21].

For CCO design, we defined the case period as the 15-day period beginning 14 days before the first event for each case. The control period was defined as the 15-day period beginning 29 days before the first event until 15 days before the first event. Cases who experienced the first event within 29 days from the start of follow-up were excluded from analysis. The effect of exposure *β*_*X*_ was approximated by estimating the conditional OR for exposure onset (i.e., the first day of *X*(*t*) = 1) in a case period versus a control period and the 95% CIs using conditional logistic regression [22].

The SSA requires cases who had experienced an exposure and event within a period of prespecified length (e.g., 15 days), irrespective of the order of exposure and event. First, a CSR was calculated by dividing the number of patients who had experienced their first event in a 15-day period beginning on the first day of exposure (i.e., exposure ➔ event) by the number of patients who had experienced their first event in a 15-day period beginning 15 days before their first exposure (i.e., event ➔ exposure). Following a previous report [20], a null-effect sequence ratio (NSR) (described in Appendix A in Additional file 1) was calculated to adjust for trends in the occurrence of either exposures or events. However, this adjustment method depends on the underlying assumption that the trends in exposures and events for the background population (i.e., all subjects included in the database) are similar to those for the study population that was used for the calculation of the CSR (e.g., the way a drug is prescribed is similar between cases and non-cases). The adjusted sequence ratio (ASR) is the ratio of CSR to NSR, and the 95% CIs were calculated based on the binomial distributions conditional on the total number of post-exposure and pre-exposure events. In Appendix B in Additional file 1, we derived CSR as a partial maximum likelihood estimator of *β*_*X*_ in a Cox model stratified by individual cases.

We calculated the bias (difference between the mean estimates and *β*_*X*_), empirical standard error (SE) of point estimates, mean squared error (MSE; sum of squared bias and empirical variance), mean estimated SE, and coverage probability of CIs (proportion of replications in which the 95% CIs included the true *β*_*X*_ value).

#### Simulation scenario for misspecification of the risk period duration

To evaluate the sensitivity of each estimator to misspecified risk period durations of exposure (*Et*_X_ = 15 days), Scenario 4 datasets with no time trends (*α*_TR_ = *β*_TR_ = 0) were analyzed by specifying erroneously short or long risk period durations (5 or 25 days, respectively).

### Case study

To compare the estimates obtained from the three self-controlled methods using real-world data, we also performed a case study that examined the risk of LI associated with the administration of macrolides using electronic medical records from the University of Tokyo Hospital (Tokyo, Japan). This electronic medical records database included information of patients who visited as outpatients or had been admitted to the hospital between January 2011 and December 2015 (approximately 244,000 patients). Data included patient characteristics (anonymized personal identifiers, age, and sex); the dates of outpatient visits, admission and discharge; prescribed medications; diagnoses; and laboratory test results.

The study population of this case study was composed of patients who had at least a 90-day continuous observation period. Each observation period was defined as the combined durations of hospitalization and consecutive outpatient visits (the intervals of hospitalization or outpatient visits were within 100 days). If the start date of hospitalization of a patient was earlier than 2011, we defined the observation start date as January 1, 2011. The observation end date was set as December 31, 2015 if a patient was still hospitalized after the end of 2015. Therefore, each patient could contribute multiple discontinuous observation periods. The exposure of interest was defined as the prescription of any macrolide approved for use in Japan, including erythromycin, roxithromycin, clarithromycin, azithromycin, spiramycin, and josamycin. The onset of LI was defined as 1) an increase in alanine aminotransferase or conjugated bilirubin that exceeded twice the upper limit of the normal range, or 2) a same-day combinatorial increase in aspartate aminotransferase, alkaline phosphatase, and total bilirubin, provided that one of these exceeded twice the upper limit of the normal range [23]. Patients who experienced the exposure or event (LI onset) within 90 days after the start date of observation were excluded.

In this case study, we designated three patterns of risk periods (15-day, 30-day, and 45-day periods) that began on the first day of macrolide prescription. These durations were based on previous findings that the onset of LI generally occurs within 1 to 6 weeks of macrolide exposure [24, 25]. In SCCS, each observation period was divided into risk periods (0–14, 0–29, or 0–44 days after exposure) and control periods (all other periods). Contiguous prescriptions of any macrolide (without an interval of one day or more of non-prescription) were considered a single risk period. Therefore, even if the patients had multiple observation periods, all these periods would contribute to the SCCS analysis. Because SCCS can simultaneously address multiple exposures, the continuous administration of macrolides within 30 days were considered a single exposure. Similarly, consecutive elevations of liver enzyme that met the definition of LI onset within 7 days were considered a single event. In CCO design, we defined a case period as 14–0, 29–0, or 44–0 days before the first LI onset; the corresponding control periods were 29–15, 59–30, or 89–45 days, respectively, before the first LI onset. In SSA, CSRs were calculated by dividing the number of patients who had experienced their first LI during 0–14, 0–29, or 0–44 days after the first day of macrolide prescription by the number of patients who had experienced their first LI during 1–15, 1–30 or 1–45 days before the first day of macrolide prescription, respectively. For the calculation of NSRs, we used the dates of the first exposure and first LI onset for each patient in the electronic medical records database (Appendix A).

Data generation of the simulation study and all statistical analyses were conducted using SAS 9.4 (SAS Institute, Cary, NC).

## Results

### Simulation study results

#### Impact of time-varying confounding

The results of simulations for Scenarios 1, 2, and 3 are presented in Tables 3, 4, and 5, respectively. Time-invariant confounders and their interactions did not introduce any bias to the point and interval estimates from all three self-controlled methods, regardless of the true IRR value (Table 3). After including transient time-varying confounding, however, we observed bias in the estimates of SCCS and CCO design. For *β*_*C*3_ = log(5.0) and *Et*_C_ = 15 days, this bias were equivalent to approximately 10% overestimation of the IRR (Table 4). The bias increased and the coverage of 95% CI fell below the nominal level (0.95) when the *Et*_C_ values increased. On the other hand, little bias was observed in SSA estimates, and the coverage of 95% CI was close to 95% for all settings of Scenario 2.

**Table 3:** Results of Simulations for Scenario 1

	Setting	Results of simulations					
Methods (Effect measures)	*β*_X_	Mean of estimates (Ratio scale)	Bias (Log scale)	Empirical standard error	Mean squared error	Mean standard error	Coverage (%)
SCCS (IRR)	log(1.0)	0.99	-0.0125	0.1354	0.0185	0.1335	95.1
CCO (OR)		1.00	-0.0005	0.2084	0.0434	0.2116	96.1
SSA (ASR)		0.99	-0.0005	0.2373	0.0563	0.2454	96.3
SCCS (IRR)	log(3.0)	2.99	-0.0030	0.0791	0.0063	0.0777	94.7
CCO (OR)		3.04	0.0121	0.18	0.0309	0.1732	94.8
SSA (ASR)		3.04	0.0119	0.2016	0.0408	0.2003	95.2
SCCS (IRR)	log(10.0)	9.98	-0.0019	0.0438	0.0019	0.0446	95.5
CCO (OR)		10.30	0.0295	0.1663	0.0285	0.1604	94.4
SSA (ASR)		9.97	-0.0026	0.1834	0.0336	0.1817	94.7

*Abbreviations ASR* adjusted sequence ratio, *CCO* case-crossover, *IRR* incident rate ratio, *OR* odds ratio, *SCCS* self-controlled case series, *SSA* sequence symmetry analysis

**Table 4:** Results of Simulations for Scenario 2

	Setting			Results of simulations					
Methods (Effect measures)	*β*_*X*_	*Et*_*C*_ (Days)^a^	*β*_*C*3_	Mean of estimates (Ratio scale)	Bias (Log scale)	Empirical standard error	Mean squared error	Mean standard error	Coverage (%)
SCCS (IRR)	log(1.0)	15	log(5.0)	1.09	0.0903	0.1540	0.0318	0.1531	88.6
CCO (OR)				1.11	0.1017	0.2336	0.0649	0.2297	93.1
SSA (ASR)				1.01	0.0145	0.2556	0.0655	0.2583	96.1
SCCS (IRR)	log(3.0)			3.32	0.1012	0.0909	0.0185	0.0890	77.0
CCO (OR)				3.35	0.1101	0.1963	0.0506	0.1894	92.3
SSA (ASR)				3.07	0.0227	0.2159	0.0471	0.2102	94.8
SCCS (IRR)	log(10.0)			11.09	0.1036	0.0507	0.0133	0.0510	46.4
CCO (OR)				11.35	0.1269	0.1774	0.0475	0.1751	91.0
SSA (ASR)				10.19	0.0193	0.1858	0.0349	0.1909	96.1
SCCS (IRR)	log(3.0)	5		3.05	0.0171	0.0946	0.0092	0.0963	94.5
CCO (OR)				3.11	0.0367	0.2012	0.0418	0.1993	94.8
SSA (ASR)				3.06	0.0213	0.2291	0.0529	0.2263	95.1
SCCS (IRR)		10		3.15	0.0501	0.0911	0.0108	0.0930	90.0
CCO (OR)				3.20	0.0643	0.1947	0.0420	0.1942	94.4
SSA (ASR)				3.09	0.0286	0.2226	0.0503	0.2193	95.1
SCCS (IRR)		20		3.50	0.1552	0.0845	0.0312	0.0852	55.0
CCO (OR)				3.54	0.1658	0.1830	0.0610	0.1849	88.5
SSA (ASR)				3.06	0.0203	0.2055	0.0426	0.2010	95.1
SCCS (IRR)		30		3.81	0.2401	0.0794	0.0639	0.0790	14.7
CCO (OR)				3.54	0.1661	0.1719	0.0571	0.1714	86.4
SSA (ASR)				3.05	0.0155	0.1916	0.0369	0.1869	94.6
SCCS (IRR)		15	log(0.2)	2.92	−0.0273	0.0977	0.0103	0.0981	95.2
CCO (OR)				2.96	−0.0129	0.2017	0.0408	0.1991	94.9
SSA (ASR)				3.06	0.0197	0.2392	0.0576	0.2316	95.1
SCCS (IRR)			log(0.5)	2.94	−0.0206	0.0972	0.0099	0.0976	95.3
CCO (OR)				2.98	−0.0058	0.2001	0.0401	0.1986	95.3
SSA (ASR)				3.04	0.0137	0.2265	0.0515	0.2295	95.4
SCCS (IRR)			log(2.0)	3.06	0.0209	0.0941	0.0093	0.0946	94.3
CCO (OR)				3.11	0.0371	0.1968	0.0401	0.1956	95.0
SSA (ASR)				3.06	0.0206	0.2249	0.0510	0.2230	94.6

*Abbreviations ASR* Adjusted sequence ratio, *CCO* Case-crossover, *IRR* Incident rate ratio, *OR* Odds ratio, *SCCS* Self-controlled case series, *SSA* Sequence symmetry analysis

^a^Length of the period in which the time-varying covariate *C*_3_(*t*) has an effect

**Table 5:** Results of Simulations for Scenario 3

	Setting			Results of simulations					
Methods (Effect measures)	*β*_*X*_	*Et*_*C*_ (Days)^a^	*β*_*C*2*C*3_	Mean of estimates (Ratio scale)	Bias (Log scale)	Empirical standard error	Mean squared error	Mean standard error	Coverage (%)
SCCS (IRR)	log(1.0)	15	log(5.0)	1.43	0.3587	0.1255	0.1445	0.1214	18.7
CCO (OR)				1.45	0.3691	0.1979	0.1754	0.1981	53.6
SSA (ASR)				1.04	0.0432	0.2104	0.0461	0.2077	94.5
SCCS (IRR)	log(3.0)			4.32	0.3656	0.0719	0.1388	0.0711	0.3
CCO (OR)				4.37	0.3762	0.1720	0.1711	0.1696	39.6
SSA (ASR)				3.12	0.0392	0.1735	0.0316	0.1704	94.5
SCCS (IRR)	log(10.0)			14.43	0.3664	0.0427	0.1361	0.0413	0.0
CCO (OR)				14.38	0.3632	0.1612	0.1579	0.1598	36.9
SSA (ASR)				10.18	0.0178	0.1589	0.0256	0.1557	95.4
SCCS (IRR)	log(3.0)	5		3.18	0.0584	0.0923	0.0119	0.0910	87.7
CCO (OR)				3.23	0.0730	0.1870	0.0403	0.1913	94.9
SSA (ASR)				3.11	0.0344	0.2224	0.0506	0.2152	94.9
SCCS (IRR)		10		3.67	0.2021	0.0803	0.0473	0.0806	30.0
CCO (OR)				3.73	0.2185	0.1799	0.0801	0.1799	79.3
SSA (ASR)				3.12	0.0385	0.1898	0.0375	0.1920	95.2
SCCS (IRR)		20		4.94	0.4978	0.0640	0.2519	0.0638	0.0
CCO (OR)				4.73	0.4562	0.1545	0.2320	0.1578	14.0
SSA (ASR)				3.09	0.0299	0.1585	0.0260	0.1531	94.2
SCCS (IRR)		30		5.85	0.6673	0.0542	0.4482	0.0543	0.0
CCO (OR)				4.51	0.4075	0.1320	0.1834	0.1323	10.5
SSA (ASR)				3.05	0.0162	0.1296	0.0171	0.1309	95.3
SCCS (IRR)		15	log(0.2)	3.06	0.0195	0.0976	0.0099	0.0947	93.7
CCO (OR)				3.08	0.0267	0.2001	0.0409	0.1949	94.6
SSA (ASR)				3.05	0.0174	0.2276	0.0521	0.2229	94.3
SCCS (IRR)			log(0.5)	3.16	0.0528	0.0944	0.0117	0.0924	89.1
CCO (OR)				3.21	0.0685	0.1979	0.0438	0.1932	94.1
SSA (ASR)				3.10	0.0340	0.2236	0.0511	0.2187	95.1
SCCS (IRR)			log(2.0)	3.61	0.1843	0.0857	0.0413	0.0833	40.4
CCO (OR)				3.67	0.2010	0.1859	0.0750	0.1838	82.7
SSA (ASR)				3.09	0.0281	0.2006	0.0410	0.1971	95.3

Abbreviations *ASR* Adjusted sequence ratio, *CCO* Case-crossover, *IRR* Incident rate ratio, *OR* Odds ratio, *SCCS* Self-controlled case series, *SSA* Sequence symmetry analysis

^a^Length of the period in which the time-varying covariate *C*_3_(*t*) has an effect

The interaction between time-varying and time-invariant confounders caused larger bias in the estimates from SCCS and CCO design (Table 5), which were equivalent to approximately 40% overestimation when interaction effect *β*_*C*2*C*3_ = log(5.0). The bias increased with increasing values of *Et*_C_ and *β*_*C*2*C*3_. These findings indicated that both SCCS and CCO design could not eliminate the interaction effect partly induced by time-invariant factors, despite being able to eliminate the main effects of these factors. However, the level of bias and CI coverage probabilities of SSA were virtually unchanged with the addition of the non-null interaction *β*_*C*2*C*3_ (Table 5).

#### Long-term time trends in exposures and events

The results of the simulations for Scenario 4 are shown in Table 6. The parameter of log(1.001) produced moderate increases in time trend, and reached an approximate 6-fold increase of the incidence rate at Day 1800 relative to the first day. In cases of exposure or event time trends, there was no substantial bias observed for all methods. However, if there were both exposure and event time trends, the SCCS estimates demonstrated bias and undercoverage of 95% CI, whereas the CCO design and SSA estimates had negligible bias.

**Table 6:** Results of Simulations for Scenario 4

	Settings		Results of simulations					
Methods (Effect measures)	*α*_TR_	*β*_TR_	Mean of estimates (Ratio scale)	Bias (Log scale)	Empirical standard error	Mean squared error	Mean standard error	Coverage (%)
SCCS (IRR)	log(1.001)	log(1.0)	3.00	0.0007	0.0579	0.0034	0.0585	95.3
CCO (OR)			3.07	0.0232	0.1213	0.0152	0.1198	94.7
SSA (ASR)			3.01	0.0044	0.1693	0.0287	0.1680	95.0
SCCS (IRR)	log(1.0)	log(1.001)	3.00	0.0006	0.0569	0.0032	0.0574	95.0
CCO (OR)			3.03	0.0111	0.1245	0.0156	0.1225	94.6
SSA (ASR)			3.01	0.0030	0.1487	0.0221	0.1464	94.7
SCCS (IRR)	log(1.001)	log(1.001)	3.79	0.2341	0.0318	0.0558	0.0314	0.0
CCO (OR)			3.07	0.0241	0.0665	0.0050	0.0676	94.4
SSA (ASR)			3.00	−0.0007	0.1053	0.0111	0.1035	94.9

*Abbreviations ASR* Adjusted sequence ratio, *CCO* Case-crossover, *IRR* Incident rate ratio, *OR* Odds ratio, *SCCS* Self-controlled case series, *SSA* Sequence symmetry analysis

#### Restricting follow-up time based on event occurrence

Although probabilistic censoring in Scenario 5 did not lead to substantial bias in the SCCS estimates, moderate undercoverage of 95% CI was observed when *P*_*C*_ was set to 1.0 (Table 7). Conceptually, the CCO design estimates would not be influenced by probabilistic censoring because the method only uses information from before the first event in each subject. On the other hand, the simulation for Scenario 5 showed severe bias and undercoverage of 95% CI in the SSA estimates.

**Table 7:** Results of Simulations for Scenarios 5 and 6

			Results of simulations					
Scenarios	Methods (Effect measures)	Settings	Mean of estimates (Ratio scale)	Bias (Log scale)	Empirical standard error	Mean squared error	Mean standard error	Coverage (%)
5	SCCS (IRR)	*P*_*C*_^a^ = 0.3	3.02	0.0055	0.1019	0.0104	0.0983	93.9
	CCO (OR)		3.02	0.0053	0.2065	0.0426	0.1974	94.2
	SSA (ASR)		4.39	0.3799	0.2764	0.2206	0.2628	72.8
	SCCS (IRR)	*P*_*C*_^a^ = 0.6	3.06	0.0202	0.1046	0.0113	0.1001	93.1
	CCO (OR)		3.02	0.0053	0.2065	0.0426	0.1974	94.2
	SSA (ASR)		7.87	0.9647	0.3638	1.0630	0.3397	0.1
	SCCS (IRR)	*P*_*C*_^a^ = 1.0	3.13	0.0421	0.1088	0.0136	0.1027	90.3
	CCO (OR)		3.02	0.0053	0.2065	0.0426	0.1974	94.2
	SSA (ASR)		NA	NA	NA	NA	NA	NA
6	SCCS (IRR)	Restricted to patients who did not experience the event before the first exposure	5.03	0.5164	0.1040	0.2775	0.1002	0.0
	CCO (OR)		3.02	0.0053	0.2065	0.0426	0.1974	94.2
	SSA (ASR)		NA	NA	NA	NA	NA	NA

*Abbreviations ASR* Adjusted sequence ratio, *CCO* Case-crossover, *IRR* Incident rate ratio, *NA* Not applicable, *OR* Odds ratio, *SCCS* Self-controlled case series, *SSA* Sequence symmetry analysis

^a^Probability of censoring upon event occurrence

#### Patient restriction based on event history

When patients who experienced the event of interest before their first exposure were excluded from the study population, we observed substantial bias and severe undercoverage of 95% CI in the SCCS estimates (Table 7) for Scenario 6. As in Scenario 5, CCO design was unaffected by patient restriction in Scenario 6 because only patients who had experienced an exposure either during the control or case periods (e.g. before the first event) contributed to this analysis.

#### Misspecification of the risk period duration

As shown in Table 8, an inadequately short risk period (5 days) led to severe bias in the CCO design estimates, but did not generate substantial bias in the SCCS and SSA estimates. While the coverage of 95% CI of SCCS and SSA maintained nominal levels (0.95), that of CCO design was close to 0%. In contrast, an excessively long risk period (25 days) produced similar bias in the estimates of all three methods (Table 8); the coverage of 95% CI for all methods were below nominal levels.

**Table 8:** Results of Simulations to Evaluate the Influence of Misspecified Risk Periods

	Settings	Results of simulations					
Methods (Effect measures)	Days of risk	Mean of estimates (Ratio scale)	Bias (Log scale)	Empirical standard error	Mean squared error	Mean standard error	Coverage (%)
SCCS (IRR)	5	2.89	−0.0377	0.1729	0.0313	0.1661	95.0
CCO (OR)		0.99	−1.1068	0.2420	1.2835	0.2406	0.5
SSA (ASR)		3.14	0.0466	0.4511	0.2056	0.4076	94.6
SCCS (IRR)	25	2.19	−0.3134	0.0907	0.1065	0.0878	4.4
CCO (OR)		2.21	−0.3056	0.1616	0.1195	0.1605	50.3
SSA (ASR)		2.23	−0.2981	0.1891	0.1246	0.1829	60.2

*Abbreviations ASR* Adjusted sequence ratio, *CCO* Case-crossover, *IRR* Incident rate ratio, *OR* Odds ratio, *SCCS* Self-controlled case series, *SSA* Sequence symmetry analysis

#### Precision of the self-controlled methods

In all the simulation scenarios, the empirical and estimated SEs of the estimates were consistently smaller in SCCS than in CCO design and SSA (Tables 3–8). As the bias in all three methods was negligible in Scenario 1 (Table 3), SSA had the highest MSE, followed by CCO design and SCCS. In Scenario 2, where SCCS produced non-negligible bias, the MSE was still smaller in SCCS than in CCO design and SSA in many settings (Table 4). However, the estimates from SCCS suffered from severe bias in Scenario 3 (Table 5). These settings resulted in a lower MSE in the SSA estimates than the other two methods, except when *Et*_C_ was set at 5 days. Similarly, if there was no considerable bias in the estimates in Scenario 4 and the simulation scenario involving the misspecification of the risk period duration, the MSE was highest for SSA, followed by CCO design and SCCS (Tables 6 and 8). In Scenarios 5 and 6, the MSE was lowest for SCCS when there was no considerable bias in estimates (Table 7).

### Case study results

Among the 114,783 patients in our electronic medical records database who had an observation period of at least 90 days, 7367 were prescribed macrolides and 14,915 patients experienced LI. The results of the case study are shown in Table 9. In the analyses of 15-day risk period, 942, 129 and 130 patients contributed the estimation of SCCS, CCO and SSA. For all different risk period durations (15, 30, and 45 days), the highest effect measure was obtained from CCO design (OR: 4.38, 3.88 and 3.23, respectively), followed by SCCS (IRR: 3.54, 2.65 and 2.23, respectively) and SSA (ASR: 2.61, 1.98 and 1.75, respectively). In our database, long-term time trends in both macrolide administration and LI were thought to be unlikely. Therefore, given a sufficient number of observed events, the SSA results would be the most reliable estimates if other drugs act as short-term time-varying confounders, interact with unmeasured genetic variants, or both. Notably, the longer the specified risk period duration, the more attenuated the relative risks that were estimated from all three methods. As shown in the simulation study with erroneously long risk periods, the longer risk periods may also bias these estimates toward lower (i.e., conservative) values. The objective of the case study was to illustrate the applications of the three self-controlled methods. Due to the fundamentally different underlying assumptions among these methods (Table 1), some of these assumptions (e.g. an event has no effect on the probability of subsequent exposure) may not be satisfied in the electronic medical records data.

**Table 9:** Results of the Case Study

Days of risk	Methods (Effect measures)	Point estimates (Ratio scale)	95% CI
15	SCCS (IRR)	3.54	3.05–4.11
	CCO (OR)	4.38	2.81–6.82
	SSA (ASR)	2.61	1.78–3.84
30	SCCS (IRR)	2.65	2.33–3.00
	CCO (OR)	3.88	2.65–5.69
	SSA (ASR)	1.98	1.44–2.72
45	SCCS (IRR)	2.23	1.99–2.51
	CCO (OR)	3.23	2.30–4.53
	SSA (ASR)	1.75	1.32–2.34

*Abbreviations ASR* Adjusted sequence ratio, *CCO* Case-crossover, *CI* Confidence interval, *IRR* Incident rate ratio, *OR* Odds ratio, *SCCS* Self-controlled case series, *SSA* Sequence symmetry analysis

## Discussion

In this study, we used large-scale simulation data to compare the effects of time-invariant and time-varying confounders, time trends in exposures and events, restricted follow-up time based on event occurrence, patient restriction based on event history, and misspecification of risk period durations on estimates among three self-controlled methods. In cases of low levels of bias in the estimates, SCCS had the lowest MSE. The estimators of CCO design were robust against censoring based on event occurrence or the exclusion of patients who experienced a pre-exposure event. The SSA estimators were less sensitive to short-term time-varying confounding, long-term time trends, and erroneously short risk periods. In contrast, the censoring of patients based on event occurrence introduced severe bias to the SSA estimates. Even in the presence of variations in the point estimates (the width of ratio-scaled differences ranged from 1.48 to 1.90) in the case study, a statistically significant increase in LI incidence was observed in the 15-, 30-, and 45-day periods after the initiation of macrolides in all three methods. The observed differences in estimates among the three methods were insufficiently large to affect decisions regarding the risk of LI due to macrolide use.

The findings that the bias in SCCS and CCO design estimates increased with the addition of the *C*_2_-*C*_3_(*t*) interaction in Scenario 2 emphasized the importance of time-invariant confounders in self-controlled methods (Table 4). The presence of such an interaction may violate the conditional Poisson modelling assumption of SCCS [14] and the conditional logistic modelling assumption of CCO design. The interaction term between *C*_2_ and *C*_3_(*t*) may not be cancelled out in the conditional likelihood equations of SCCS and CCO design because the distributions of *C*_3_(*t*) are unbalanced between the risk/case periods and the control periods. When time-varying confounders modify the effect of time-invariant factors on an outcome (e.g., when a transient infectious disease modifies the effect of genetics as a trigger of liver disease), or vice versa, the influence of these factors would be non-negligible in SCCS and CCO design.

As shown in Appendix B, the estimator from the SSA method can essentially be considered a hazard ratio estimator from a Cox model stratified at the patient level. An important condition of the proportionality of hazards between the exposed and non-exposed periods is that any covariate that affects event occurrence must be invariant during the exposed and non-exposed periods (i.e., a time-constant baseline hazard). If this condition is not met, the estimator would suffer from bias due to model misspecification (although proportionality of non-constant hazards is possible in theory, it is unlikely in practice). In our simulation study, however, these possible misspecification effects are likely to be minimal because 1) the time-varying confounder *C*_3_(*t*) rarely changed status (0 to 1 or 1 to 0) during the 30-day period, and 2) even if *C*_3_(*t*) changed status during the 30-day period, the days of *C*_3_(*t*) = 1 are likely to be balanced between the pre- and post-exposure periods. Since this simulation data are considered to be realistic, the SSA method is expected to be robust to the effects of similar types of unmeasured time-varying confounding. *C*_3_(*t*) may affect exposure during *Et*_C_ days after its onset, and its effect may be nullified after *Et*_C_ days had elapsed. This means that the onset days of *C*_3_(*t*) were uniformly distributed over *Et*_C_ days before exposure, and that the days in which the effect ended were also uniformly distributed over the same period. Therefore, even if *C*_3_(*t*) affected exposure, the distribution of the days of *C*_3_(*t*) = 1 would be the same between *Et*_C_ days before and after exposure onset, which in turn would nullify the bias arising from *C*_3_(*t*) (Tables 4 and 5). Hence, even if a time-varying covariate (e.g., an acute condition) leads to both higher exposure and event probabilities, this would simply imply that there are more periods with exposure and events. Consequently, this would not be expected to affect SSA estimates, which are essentially based on a “what comes first” comparison. Although the SSA method is robust to this type of unmeasured time-varying confounding, it may be affected when the magnitude of time-varying confounding changes gradually over time.

As shown in Table 5, the estimates of SCCS were considerably biased in the presence of time trends in *both* exposure and event onset. This finding was not consistent with the results of a previous simulation study that the SCCS method was relatively robust to time trends in both exposures and events [12]. This discrepancy may be partially explained by the previous study’s use of shorter observations (500 days compared to 1800 days in our study), lower baseline incidence (2*10^−7^, which is 100 times smaller than our baseline rate), and lack of consideration to multiple exposures. In contrast, the estimates of CCO design and SSA in our study were less sensitive to the time trends in exposure and event onset (Table 6) because these two methods analyze short-term periods (30 days for each patient). Although previous studies showed that exposure time trends introduced bias to CCO design estimates [16, 17], the magnitude of bias would not be considerable for moderate time-trend effects (as set in this study). Moreover, since the time trends in exposures or events were constant for all patients in Scenario 3, NSR could be used to adjust for the effects of these trends on SSA estimates.

The severe overestimation of the SSA estimates in Scenario 5 may be the manifestation of selection bias due to the probabilistic censoring of some patients who would experience their first exposure after the first event (Table 7), which led to a reduction in the denominator for CSR calculation. This indicates that the SSA method may be unsuitable for situations where patients could be censored due to the event of interest (e.g. myocardial infraction or intracerebral hemorrhage). In contrast, SCCS estimators were less sensitive to patient restriction even when all patients were censored at the occurrence of their first event. However, SCCS estimators could become severely biased in Scenario 6 (Table 7) when patients who had experienced the event before their first exposure were excluded from the study population. It should be noted that these events have no effect on the probabilities of future exposures or events occurrence in this scenario. These findings suggest that among the three methods, CCO design may be suitable for analyses of events that have a possible effect on the occurrence of future exposures or events.

The specification of risk periods of exposure is crucial to all three methods, but different indications can be applied. For example, erroneously short periods introduced bias only to CCO design estimates, whereas erroneously long risk periods introduced bias to the estimates from all methods (Table 8). If erroneously short risk periods are specified in CCO design, a section of the case periods that have a higher likelihood of exposure would be incorrectly classified as control periods. Although similar misclassifications (true risk periods incorrectly defined as control periods) can also occur for SCCS, these would not cause considerable bias because the control periods are substantially longer than the risk periods. There was a lack of such misclassifications in SSA because this method compares the incidence rate between pre- and post-exposure periods. In contrast to erroneously long risk periods, there were misclassifications where control periods were incorrectly defined as risk/case periods for all methods, which led to an over-specification of the risk period (Table 8). Since the overall duration of risk periods was substantially shorter than that of control periods in SCCS, the merging of control periods with risk periods biased the estimates toward lower values. Similarly, such misclassification of risk periods also caused underestimations in CCO design and SSA because these two methods compared the incidence between two periods of the same length.

This study has several limitations. First, the scenarios used were restricted to transient exposure effects, had only a few confounders, no censoring, and no effect modification. Nevertheless, the relatively simple scenarios examined here were suitable for facilitating an understanding of the lesser-known effects of these factors on each estimation method. Second, we did not evaluate multiple definitions of the various periods used in the three methods. For example, the risk periods of SCCS can be divided into more than one interval [11], and multiple control periods can be set in CCO designs [7]. However, because the observed bias for SCCS and CCO design could be explained by the asymmetrical distribution of *C*_3_(*t*), this bias would not be eliminated by changing the definition of the periods without further information on the covariates. Finally, because the electronic medical records used in this case study were obtained from one university hospital, the exposures and events that occurred outside of the hospital (such as at a patient’s home or other clinics) could not be recorded.

## Conclusion

Our simulation study showed that the estimations of SCCS, CCO design and SSA depended on various underlying assumptions, and that the violation of these assumptions could lead to non-negligible bias in the resulting estimates. All three methods indicated that macrolide use was significantly associated with an increased risk of LI in the case study, despite small variations in estimates across the methods. Pharmacoepidemiologists should select the appropriate self-controlled method based on how well the relevant key assumptions are satisfied with respect to the available data. Moreover, if several self-controlled methods produce different estimates for the same data, analysts should carefully consider the presence of factors that may have introduced bias to the estimates.

## Additional files

Additional file 1: Appendix A. Calculation of the null-effect sequence ratio. Appendix B. Equivalence between the crude sequence ratio and the incidence rate ratio estimator calculated using a stratified Cox model. Appendix C. SAS program codes of the simulation study. (DOCX 67 kb)

## Abbreviations

ASR: Adjusted sequence ratio
CCO: Case-crossover
CI: Confidence interval
CSR: Crude sequence ratio
IRR: Incidence rate ratio
LI: Liver injury
MSE: Mean squared error
NA: Not applicable
NSR: Null-effect sequence ratio
OR: Odds ratio
SCCS: Self-controlled case series
SE: Standard error
SSA: Sequence symmetry analysis
