# Supplementary material for: A comparison of estimators from self-controlled case series, case-crossover design, and sequence symmetry analysis for pharmacoepidemiological studies
Source: BMC Med Res Methodol. 2018 Jan 8;18:4. doi: 10.1186/s12874-017-0457-7 (PMC5759844; doi:10.1186/s12874-017-0457-7)
Supplement: Supplementary file 1 — Appendix A. Calculation of the null-effect sequence ratio. Appendix B. Equivalence between the crude sequence ratio and the incidence rate ratio estimator calculated using a stratified Cox model. Appendix C. SAS program codes of the simulation study. (DOCX 67 kb) [file 12874_2017_457_MOESM1_ESM.docx]

**Additional file 1**

# **Appendix A. Calculation of the null-effect sequence ratio**

A null-effect sequence ratio (NSR) can be interpreted as the odds ratio of the exposure occurring prior to the event for the source population of a sequence symmetry analysis [1, 2]. This ratio assumes that the exposure and event occur a maximum of one time throughout the study period for each patient. *E_m_* denotes the number of exposures that occurred on day *m* and *O_n_* denotes the number of events that occurred on day *n*. When the length of the period with a risk of exposure is defined as *k* days, the crude sequence ratio is calculated by dividing the number of subjects who had experienced the first event at 0 to *k*-1 days after exposure (i.e., exposure 🡪 event) by the number of subjects who had experienced the event at 1 to *k* days before exposure (i.e., event 🡪 exposure). Subsequently, the number of exposure-event combinations where the event occurred 0 to *k*-1 days after exposure in the source population *N_EO_* was obtained using the equation,

$N_{EO}=\sum_{m=1}^{u} E_{m}\left(\sum_{n=m}^{\min\{u,m+k-1\}} O_{n}\right)$,

where *u* is the end date of the study period. Note that the exposure and event do not necessarily occur in the same patient for each combination. Similarly, the number of exposure-event combinations where the event occurred 1 to *k* days before exposure in *N_OE_* was obtained using the equation,

$N_{OE}=\sum_{m=1}^{u} E_{m}\left(\sum_{n=\max\{0,m-k\}}^{m-1} O_{n}\right)$.

Note that *O_0_* is zero. The proportion of exposure-event combinations where the event occurred 0 to *k*-1 days after exposure to the exposure-event combinations where the event occurred *k* days before to *k*-1 days after exposure in the source population *P_A_* was obtained using the equation,

$P_{A}=\frac{N_{EO}}{N_{EO}+N_{OE}}=\frac{\sum_{m=1}^{u} E_{m}\left(\sum_{n=m}^{\min\{u,m+k-1\}} O_{n}\right)}{\sum_{m=1}^{u} E_{m}\left(\sum_{n=\max\left\{0,m-k\right\}}^{\min\left\{u,m+k-1\right\}} O_{n}\right)}$.

Therefore, the NSR was then calculated using the equation,

$\text{ NSR}=\frac{P_{A}}{1-P_{A}}=\frac{N_{EO}}{N_{OE}}$.

Note that the patients who experienced an exposure, an event, or both in each observation period only contributed to the calculation of NSR.

In this study, we set the parameters of *u* = 1,800 (days) and *k* = 15 (days). Thus, NSR was calculated using the equation,

$\text{NSR}=\frac{\sum_{m=1}^{1800} E_{m}\left(\sum_{n=m}^{\min\{1800,m+14\}} O_{n}\right)}{\sum_{m=1}^{1800} E_{m}\left(\sum_{n=\max\left\{0,m-15\right\}}^{m-1} O_{n}\right)}$.

# **Appendix B. Equivalence between the crude sequence ratio and the incidence rate ratio estimator calculated using a stratified Cox model**

The risk and control periods were defined as 15-day periods after and before exposure, respectively. Consider a Cox model stratified by each patient

*λ_i_*(*t*| Risk period) = *λ_i_*(*t*| Control period)exp(*β*),

where *λ_i_*(*t*|⋅) is an unspecified hazard on day *t* after the start of the risk or control periods of patient *i*, and *β* is a common log-hazard ratio across patients that is constant throughout time. Letting *p* denote the indicator for the risk period (*p* = 1; 0 for control), the model is rewritten as *λ_ip_*(*t*) = *λ_i_*_0_(*t*)exp(*βp*), where *λ_ip_*(*t*) is the hazard on day *t* in period *p* of patient *i*. By stratification, patients are treated as “matched” pairs of risk and control periods, and the stratified Cox model removes time-invariant confounding across individuals if the modeling assumption holds.

The hazard ratio estimator was obtained by maximizing partial likelihood,

$L(\beta)=\prod_{i=1}^{100,000} \prod_{p=0,1} \left(\frac{\exp(\beta p)}{\sum_{p'=0,1\text{ if remained in patient }i\text{'s risk set at }T_{ip}} \exp(\beta p')}\right)^{D_{ip}}$,

where observed time *T_ip_* is the minimum number of days from the start of the period to event occurrence and 15 days, and *D_ip_* is an event indicator in period *p* of patient *i* [3]. It can be shown that in pair-matched data, the pairs that contribute to the partial likelihood are restricted to ones in which a member (i.e., period) with shorter observed time has experienced an event. The maximum partial likelihood estimator of hazard ratio exp(*β*) is the ratio of (a) the number of pairs in which the exposed member (*p* = 1) has a shorter observed time and experienced an event and (b) the number of pairs in which the non-exposed member (*p* = 0) has a shorter observed time and experienced an event [3].

By applying this to our sequence symmetry analysis method, the patients that contribute to the partial likelihood *L*(*β*) are the ones who experienced an event in either period. (Because our sequence symmetry analysis method restricts events and exposures to their first occurrence, patients cannot experience an “event” in both the risk and control periods. Furthermore, we assumed that no censoring occurred within the 15-day risk and control periods.) Thus, the ratio of the number of events in the risk period and the number of events in the control period (i.e., crude sequence ratio) is the maximum partial likelihood estimator of hazard ratios for the risk and control periods, which is equal to the incidence rate ratio under constant hazards, i.e., *λ_ip_*(*t*) = *λ_ip_*.

# **Appendix C. SAS program codes of the simulation study**

*R_SCM_simulation.sas*

/* Note: This SAS program is used to set the parameters of each simulation and to run SAS macro program “%scm_simulation”. Before running this program, please store SAS program files “*M_SCM_simulation.sas*” (described below) as well as “*sccs.sas*”, “*element.sas*” and “*poisreg.sas*”, which available from the Open University website, UK <http://statistics.open.ac.uk/sccs/index.htm>, in your macro directory defined as &macdir*/

options nosource nonotes;

ods listing close;

%LET macdir =;

%LET outdir =;

%LET indir =;

%INCLUDE "&macdir\sccs.sas";

%INCLUDE "&macdir\element.sas";

%INCLUDE "&macdir\poisreg.sas";

%INCLUDE "&macdir\M_SCM_simulation.sas";

/*Definition of macro variables for *%scm_simulation**/

/*Length of days which *C*_3_(*t*) has effect (*E_tC_*)*/

%let tcvrg = 15;

/*Parameters for exposure *X*(*t*) generation*/

%let fai0 = log(1/4000); /*Baseline incidence of exposure (fixed)*/

%let fai1 = log(2.0); /*Effect of *C*1 (fixed)*/

%let fai2 = log(1.2); /*Effect of *C*2 (fixed)*/

%let fai3 = log(5.0); /*Effect of *C*_3_(*t*) (fixed)*/

/*Parameters for event *Y* generation*/

%let beta0 = log(0.00002); /*Baseline incidence of event (fixed)*/

%let beta1 = log(3.0); /*Effect of exposure *X*(*t*) *β_X_**/

%let beta2 = log(2.0); /*Effect of *C*_1_ *β_C_*_1_ (fixed)*/

%let beta3 = log(3.0); /*Effect of *C*_2_ *β_C_*_2_ (fixed)*/

%let beta4 =; /*Effect of *C*_3_(*t*) *β_C_*_3_*/

%let beta5 =; /*Interaction effect of *C*_1_**C*_2_ *β_C_*_1_*_C_*_2_*/

%let beta6 =; /*Interaction effect of *C*_2_**C*_3_(*t*) *β_C_*_2_*_C_*_3_*/

/*Parameters for time-trend*/

%let trend = N; /*If trend=Y, the *X*(*t*) and *Y* were generated by Equations 3 and 4, respectively*/

%let fai4 =; /*Time-trend of exposure generation *α_TR_**/

%let beta7 =; /*Time-trend of event generation *β_TR_**/

/*Length of risk period for analysis*/

%let rkrg = 15;

/*Start No. of iteration (fixed)*/

%let rpst = 1;

/*Probability of censoring at event time *P_C_**/

%let pcens = 0.0;

/*If &excld1 = Y, the patients who experienced event before their first exposure were excluded*/

%let excld1 = N;

%***scm_simulation*** (nroop=**2000**, N=**1800**, Numsamples=**100000**);

/*nroop: Last no. of iteration (fixed)*/

/*N: Days of observation (fixed)*/

/*Numsamples: No. of patients (fixed)*/

options source notes;

ods listing;

*M_SCM_simulation.sas*

/* Note: This SAS program includes SAS macro program “%scm_simulation” used for this simulation study. Before conducting this simulation, please store this program file as well as “*sccs.sas*”, “*element.sas*” and “*poisreg.sas*”, which available from the Open University website, UK <http://statistics.open.ac.uk/sccs/index.htm>, in your macro directory defined in *R_SCM_simulation.sas**/

**%MACRO** scm_simulation (nroop=, N=, Numsamples=);

proc datasets;

delete sccs_result cco_result ssa_result;

quit;

%DO roop= &rpst %TO &nroop;

%let seed1 = (&roop*12345);

%let seed2 = (&roop*100000);

/*Data generation step (no time-trend of exposure or event)*/

%IF &trend = N %THEN %DO;

/*Generation of covariates*/

data scs01;

call streaminit(&seed1);

do ID = **1** to &NumSamples;

retain scov1 scov2 cumday;

scov1 = rand("Bernoulli", **0.05**);

scov2 = rand("Bernoulli", **0.5**);

cumday = **1**;

do i = **1** to &N;

tcvs1 = rand("Exponential",**1000**);

cumday + tcvs1;

output;

if cumday >= (&N+**1**) then leave;

end;

end;

run;

/*Connection of the periods during which *C*_3_(*t*) has effect within single day*/

data scs02;

set scs01;

if cumday >= (&N+**1**) then delete;

tcvs2 = int(cumday);

tcve2 = tcvs2 + (&tcvrg-**1**);

run;

proc sort data=scs02 nodupkey;

by id tcvs2;

run;

data scs03(keep=id scov1 scov2 tcvs2 tcve2 pd_id);

set scs02; by id tcvs2;

retain pd_id;

if first.id then pd_id=**0**;

if first.tcvs2 then do;

pd_id=pd_id+**1**;

end;

run;

data scs04(keep=id pd_id scov1 scov2 tcvs2 tcve2 n_dur s_unite);

set scs03; by id;

le_day=lag1(tcve2);

if first.id then le_day=tcvs2;

n_dur=tcvs2-le_day-**1**;

s_unite=(n_dur=<**0**);

run;

data scs05(keep=id pd_id scov1 scov2 tcvs2 tcve2 s_unite n_dur npd_id);

set scs04; by id;

retain npd_id;

if first.id then npd_id=**1**;

npd_id=npd_id+(**1**-s_unite);

run;

data scs06(keep=id npd_id scov1 scov2 tcvs3 tcve3);

set scs05; by id npd_id;

retain npd_id tcvs3;

if first.npd_id then tcvs3 = tcvs2-**1**;

if last.npd_id then do;

tcve3 = tcve2;

output;

end;

run;

/*Import of max No. of time-varying covariate *C*_3_(*t*) for each patient*/

proc sql;

select max(npd_id) into :maxtcov

from scs06;

quit;

proc transpose data=scs06 out=scs07 (drop=_NAME_) prefix=tcvs;

var tcvs3;

by id;

run;

data scs08;

set scs06;

tcve4 = min(&N, tcve3);

run;

proc transpose data=scs08 out=scs09 (drop=_NAME_) prefix=tcve;

var tcve4;

by id;

run;

/*Create of dataset of 1 record per interval*/

data scs10;

merge scs07 scs09;

by id;

begin = **0**;

end = &N;

run;

data scs11;

set scs10;

array x(*) begin end %DO i=**1** %TO &maxtcov; tcvs&i tcve&i %END;;

do i=**1** to DIM(x);

if x[i] ne **.** and x[i] >= begin and x[i] <= end then do;

stop = x[i];

output;

end;

end;

run;

proc sort data=scs11 out=scs12;

by id stop;

run;

data scs13;

retain id stop offset tcov1;

set scs12;

by id;

start = lag(stop);

if first.id then start = begin;

if start=stop then delete;

offset=(stop-start);

%DO i=**1** %TO &maxtcov;

tcv&i = (start = tcvs&i and stop= tcve&i);

%END;

tcov1=**0**

%DO i=**1** %TO &maxtcov;

+ tcv&i

%END;;

;

run;

data scs14(keep=id scov1 scov2);

set scs01;

run;

proc sort data=scs14 nodupkey;

by id;

run;

proc sql;

create table scs15 as

select %DO i=**1** %TO &maxtcov; b.tcvs&i, b.tcve&i, %END; b.start, b.stop, b.tcov1, a.scov1, a.scov2, a.id

from scs14 a left join scs13 b on a.ID=b.ID;

quit;

data scs16;

set scs15;

if start=**.** then do;

start=**0**;

stop=&N;

tcov1=**0**;

end;

run;

proc sort data=scs16 out=scs17;

by id start;

run;

/*Generation of exposures*/

data scs18;

call streaminit(&seed1);

set scs17;

by id start;

retain cumday;

cumday = start + **1**;

do i = **1** to &N;

exps1 = rand("Exponential", (**1**/exp(&fai0 + &fai1*scov1 + &fai2*scov2 + &fai3*tcov1)));

cumday + exps1;

output;

if cumday >= (stop+**1**) then leave;

end;

run;

/*Connection of the risk periods within single day*/

data scs19 (keep= id exps2 expe2);

set scs18;

if cumday >= (stop+**1**) then delete;

exps2=int(cumday);

expe2=exps2+**14**;

run;

proc sort data=scs19 nodupkey;

by id exps2;

run;

data scs20(keep=id exps2 expe2 pd_id);

set scs19; by id exps2;

retain pd_id;

if first.id then pd_id=**0**;

if first.exps2 then do;

pd_id=pd_id+**1**;

end;

run;

data scs21(keep=id pd_id exps2 expe2 n_dur s_unite);

set scs20; by id;

le_day=lag1(expe2);

if first.id then le_day=exps2;

n_dur=exps2-le_day-**1**;

s_unite=(n_dur=<**0**);

run;

data scs22(keep=id pd_id exps2 expe2 s_unite n_dur npd_id);

set scs21; by id;

retain npd_id;

if first.id then npd_id=**1**;

npd_id=npd_id+(**1**-s_unite);

run;

data scs23(keep=id npd_id exps3 expe3);

set scs22; by id npd_id;

retain npd_id exps3;

if first.npd_id then exps3 = exps2-**1**;

if last.npd_id then do;

expe3 = expe2;

output;

end;

run;

/*Import of max No. of exposure *X*(*t*) for each patient*/

proc sql;

select max(npd_id) into :maxexp

from scs23;

quit;

proc transpose data=scs23 out=scs24 (drop=_NAME_) prefix=exps;

var exps3;

by id;

run;

data scs25;

set scs23;

expe4 = min(&N, expe3);

run;

proc transpose data=scs25 out=scs26 (drop=_NAME_) prefix=expe;

var expe4;

by id;

run;

data scs27;

merge scs24 scs26;

by id;

run;

proc sql;

create table scs28 as

select %DO i=**1** %TO &maxtcov; b.tcvs&i, b.tcve&i, %END; a.scov1, a.scov2, a.id

from scs14 a left join scs10 b on a.ID=b.ID;

quit;

proc sql;

create table scs29 as

select %DO i=**1** %TO &maxexp; b.exps&i, b.expe&i, %END; a.*

from scs28 a left join scs27 b on a.ID=b.ID;

quit;

/*Create of dataset of 1 record per interval*/

data scs30;

set scs29;

begin=**0**;

end=&N;

array x(*) begin end %DO i=**1** %TO &maxtcov; tcvs&i tcve&i %END; %DO j=**1** %TO &maxexp; exps&j expe&j %END;;

do i=**1** to DIM(x);

if x[i] ne **.** and x[i] >= begin and x[i] <= end then do;

stop = x[i];

output;

end;

end;

run;

proc sort data=scs30 out=scs31;

by id stop;

run;

data scs32;

retain id stop offset tcov1 rx1;

set scs31;

by id;

start = lag(stop);

if first.id then start = begin;

if start=stop then delete;

offset=(stop-start);

%DO i=**1** %TO &maxtcov;

tcv&i = (start => tcvs&i and stop <= tcve&i);

%END;

%DO j=**1** %TO &maxexp;

exp&j = (start => exps&j and stop <= expe&j);

%END;

tcov1=**0**

%DO i=**1** %TO &maxtcov;

+ tcv&i

%END;;

rx1=**0**

%DO j=**1** %TO &maxexp;

+ exp&j

%END;;

;

run;

/*Generation of events*/

data scs33;

call streaminit(&seed2);

set scs32;

by id start;

retain cumday;

cumday = start + **1**;

do i = **1** to &N;

out1 = rand("Exponential",(**1**/exp(&beta0 + &beta1*rx1 + &beta2*scov1 + &beta3*scov2 + &beta4*tcov1 + &beta5*scov1*scov2 + &beta6*scov2*tcov1)));

cumday + out1;

output;

if cumday >= (stop+**1**) then leave;

end;

run;

data scs34 (keep=id out2);

set scs33;

if cumday >= (stop+**1**) then delete;

out2 = int(cumday);

run;

/*Import of max No. of event *Y* for each patient*/

data sum01;

set scs34;

out2=**1**;

run;

proc univariate data=sum01;

by ID;

var out2;

output out=sum02 sum=tout;

run;

proc sql;

select max(tout) into :maxevent

from sum02;

quit;

proc transpose data=scs34 out=scs35 (drop=_NAME_) prefix=event;

var out2;

by id;

run;

proc sql;

create table scs36 as

select %DO i=**1** %TO &maxevent; b.event&i, %END; a.*

from scs29 a left join scs35 b on a.ID=b.ID;

quit;

/*Probabilistic censoring at event time*/

data scs34x;

call streaminit(&seed2);

set scs34;

censor = rand("Bernoulli", &pcens);

run;

data scs34xx(keep=id cday);

set scs34x;

if censor=**1**;

cday=out2;

run;

proc sort data=scs34xx;

by id cday;

run;

data scs34xxx;

set scs34xx;

by id;

if first.id;

run;

/*Using of the exposure date before connection*/

data scs100;

set scs19;

by id;

retain exp_id;

if first.id then exp_id=**0**;

exp_id=exp_id+**1**;

run;

proc sql;

select max(exp_id) into :maxexp2

from scs100;

quit;

proc transpose data=scs100 out=scs101 prefix=exp;

by id;

var exps2;

run;

proc sql;

create table scs36x as

select %DO i=**1** %TO &maxexp2; b.exp&i, %END; a.*

from scs36 a left join scs101 b on a.ID=b.ID;

quit;

proc sql;

create table scs36xx as

select a.*, b.cday

from scs36x a left join scs34xxx b on a.ID=b.ID;

quit;

data scs37 (keep= id %DO i=**1** %TO &maxexp2; exp&i %END; %DO j=**1** %TO &maxevent; event&j %END; startobs endobs dob);

set scs36xx;

startobs=**1**;

endobs= min(&N, cday);

dob=**0**;

if event1=**.** then delete;

/*If &excld1 = Y, the patients who experienced event before their first exposure were excluded*/

%IF &excld1 = Y %THEN %DO;

if event1 < exp1 then delete;

%END;

run;

%END;

/*End of data generation step (no time-trends of exposure and event)*/

/*Data generation step (there are time-trends of exposure and event)*/

/*Note: Data generation process using a piecewise exponential distribution was not available if time-trends of exposure and event were included in rate parameters for data generation.

Therefore, in the presence of such time-trends (&trend=Y), *C*_3_(*t*), *X*(*t*) and *Y* were generated using Bernoulli distributions day by day although longer computational time was needed.*/

%IF &trend=Y %THEN %DO;

/*Generation of covariates*/

data scs01;

call streaminit(&seed1);

do ID = **1** to &NumSamples;

retain scov1 scov2 tcov1;

scov1 = rand("Bernoulli", **0.05**);

scov2 = rand("Bernoulli", **0.5**);

do day = **1** to &N;

tcov1 = rand("Bernoulli", **0.001**);

output;

end;

end;

run;

data scs02;

set scs01;

retain tcved;

by ID;

if first.id then tcved=**0**;

if tcov1=**1** then tcved=min(day+(&tcvrg-**1**), &N);

run;

data scs03(drop=tcved);

set scs02;

if day =< tcved then tcov1=**1**;

run;

data scs04 exp01(keep=id day exp);

call streaminit(&seed1);

set scs03;

exp = rand ("Bernoulli", exp(&fai0 + &fai1*scov1 + &fai2*scov2 + &fai3*tcov1 + &fai4*day));

output scs04;

if exp=**1** then output exp01;

run;

data scs05;

set scs04;

retain exped;

by ID;

if first.id then exped=**0**;

if exp=**1** then exped=min(day+**14**, &N);

run;

data scs06(drop=exped);

set scs05;

if day =< exped then exp=**1**;

run;

data event01(keep=id day event);

call streaminit(&seed2);

set scs06;

event = rand ("Bernoulli", exp(&beta0 + &beta1*exp + &beta2*scov1 + &beta3*scov2 + &beta4*tcov1 + &beta5*scov1*scov2 + &beta6*scov2*tcov1 + &beta7*day));

if event=**1** then output;

run;

/*Import of max No. of time-varying covariate *C*_3_(*t*) for each patient*/

data exp02;

set exp01;

retain numexp;

by id;

if first.id then numexp=**0**;

numexp=numexp+**1**;

run;

proc sql;

select max(numexp) into :maxexp2

from exp02;

quit;

proc transpose data=exp02 out=exp03(drop=_NAME_) prefix=exp;

var day;

by id;

run;

/*Import of max No. of exposure *X*(*t*) for each patient*/

data event02;

set event01;

retain numevt;

by id;

if first.id then numevt=**0**;

numevt=numevt+**1**;

run;

proc sql;

select max(numevt) into :maxevent

from event02;

quit;

proc transpose data=event02 out=event03(drop=_NAME_) prefix=event;

var day;

by id;

run;

data scs36xx;

merge exp03 event03;

by id;

run;

data scs37;

set scs36xx;

startobs=**1**;

endobs=&N;

dob=**0**;

if event1=**.** then delete;

run;

%END;

/*End of data generation step (there are time-trends of exposure and event)*/

/*Analysis by SCCS*/

/*SAS programs “*element.sas*”, “*sccs.sas*” and “*poisreg.sas*” were available from the Open University website, UK

<http://statistics.open.ac.uk/sccs/index.htm>.*/

/*Definition of macro variables for *%sccs**/

%LET agerange=1 &N;

%LET risk=0 (&rkrg-1);

%LET age=;

%LET season=;

%LET semi=N;

/*Create of dataset for poireg.sas*/

%***sccs***(data=scs37, dob_raw=dob, pid=id, events= %DO i=**1** %TO &maxevent; event&i %END;, vacc=%DO i=**1** %TO &maxexp2; exp&i %END;, startst=startobs, endst=endobs, outdata=sccs01);

/*Estimation of IRR*/

%***poisreg***(data=sccs01,y=nevt,covar=int riskr1,offset=offset,elim=ID,prntyn=N,title="SCM simulation");

data out2(keep= estimate sterr ll ul expest expll expul);

set out;

where params like '%RISK%';

run;

proc append base=sccs_result data=out2;

run;

/*Analysis by CCO design*/

/*Definition of risk and control periods*/

data scs38(keep=id %DO i=**1** %TO &maxexp2; exp&i %END; event1 contst conted casest caseed end);

set scs37;

if event1 < (**2***&rkrg) then delete;

end=min(&N, event1);

contst=event1 - ((**2***&rkrg)-**1**);

conted=event1 - &rkrg;

casest=event1 - (&rkrg-**1**);

caseed=event1;

run;

/*Determination of exposure for risk and control periods*/

data scs39;

set scs38;

%DO i=**1** %TO &maxexp2;

cont&i = (contst <= exp&i and exp&i <= conted);

%END;

%DO j=**1** %TO &maxexp2;

case&j = (casest <= exp&j and exp&j <= caseed);

%END;

cont=**0**

%DO i=**1** %TO &maxexp2;

+ cont&i

%END;;

case=**0**

%DO j=**1** %TO &maxexp2;

+ case&j

%END;;

run;

data scs40(keep=id exp);

set scs39;

exp=**1**;

if case => **1** then output;

run;

proc sort data=scs40 nodupkey;

by ID;

run;

proc sql;

create table scs41 as

select a.id, b.exp

from scs38 a left join scs40 b on a.ID=b.ID;

quit;

data scs42;

set scs41;

period=**1**;

if exp=**.** then exp=**0**;

run;

data scs43(keep=id exp);

set scs39;

exp=**1**;

if cont => **1** then output;

run;

proc sort data=scs43 nodupkey;

by ID;

run;

proc sql;

create table scs44 as

select a.id, b.exp

from scs38 a left join scs43 b on a.ID=b.ID;

quit;

data scs45;

set scs44;

period=**0**;

if exp=**.** then exp=**0**;

run;

data cco01;

set scs42 scs45;

run;

proc sort data=cco01 nodup;

by id period;

run;

/*Estimation of OR*/

proc logistic data=cco01 outest=out3 covout descending;

strata ID;

model period=exp;

run;

proc transpose data=out3 out=out4 (drop=_NAME_) prefix=param;

var exp;

run;

data out5 (keep=estimate sterr ll ul expest expll expul);

set out4;

estimate=param1;

sterr=sqrt(param2);

ll=param1-**1.96***sqrt(param2);

ul=param1+**1.96***sqrt(param2);

expest=exp(estimate);

expll=exp(ll);

expul=exp(ul);

run;

proc append base=cco_result data=out5;

run;

/*Analysis by SSA*/

data scs46x (keep= id exp event cday);

set scs36xx;

exp=exp1;

event=event1;

if cday=**.** then cday=&N;

if exp=**.** and event=**.** then delete;

run;

data scs46 (drop=cday);

set scs46x;

if exp > cday then exp=**.**;

/* If &excld1 = Y, the patients who experienced event before their first exposure were excluded*/

%IF &excld1 = Y %THEN %DO;

if event < exp then delete;

%END;

run;

data scs47;

set scs46;

if exp ne **.** and event ne **.** then both=**1**;

else both=**0**;

run;

/*If exposure or event was null, the next day of end of observation (e.g. 1801) was substituted*/

data scs48;

set scs47;

if exp=**.** then exp= &N+**1**;

if event=**.** then event= &N+**1**;

run;

/*Calculation of the days between exposure and event*/

data scs49(keep=id exp event rd_drg rd_dia both);

set scs48;

prev=**0**;

RD_DRG=exp-prev;

RD_DIA=event-prev;

run;

data scs50(keep=id rd_drg rd_dia both DIF DIA_Gx_DRG flag);

set scs49;

flag=**1**;

if both=**1** then do;

DIF=rd_dia-rd_drg;

DIA_Gx_DRG=(rd_drg=<rd_dia);

end;

run;

proc sort data=scs50; by rd_drg rd_dia;

run;

/*Extraction of patients who contributed the calculation of CSR*/

data scs51(keep= id rd_drg rd_dia both DIA_Gx_DRG);

set scs50;

if -(&rkrg)<=DIF=<(&rkrg-**1**) then output;

run;

proc means data=scs51 n sum noprint;

where both=**1**;

var DIA_Gx_DRG;

output out=ssa_c01 n=TOTAL sum=DIA_Gx_DRG;

run;

/*Calculation of marginal distribution for exposure and event*/

data ssa_c02(keep= DRG1-DRG&N DIA1-DIA&N s_DRG s_DIA s_Upper s_Diago s_Lower flag);

set scs50;

by flag;

array DRG(*) DRG1-DRG&N; array DIA(*) DIA1-DIA&N;

retain DRG1-DRG&N DIA1-DIA&N;

if first.flag then do;

do i=**1** to &N; DRG(i)=**0**; DIA(i)=**0**; end;

end;

if rd_drg<(&N+**1**) then DRG(rd_drg)=DRG(rd_drg)+**1**;

if rd_dia<(&N+**1**) then DIA(rd_dia)=DIA(rd_dia)+**1**;

if last.flag then do;

s_DRG=**0**; do i=**1** to &N; s_DRG=s_DRG+DRG(i); end;

s_DIA=**0**; do j=**1** to &N; s_DIA=s_DIA+DIA(j); end;

/*No. of exposure-event combinations where the event occurred after exposure in the source population */

s_Upper=**0**;

do i=**1** to (&N-**1**);

js=i+**1**;

do j=js to &N;

if **0**<j-i<=(&rkrg-**1**) then s_Upper=s_Upper+DRG(i)*DIA(j); end;

end;

/*No. of exposure-event combinations where the event and exposure occurred in same day in the source population */

s_Diago=**0**;

do i=**1** to &N; s_Diago=s_Diago+DRG(i)*DIA(i); end;

/*No. of exposure-event combinations where the event occurred before exposure in the source population */

s_Lower=**0**;

do j=**1** to (&N-**1**);

is=j+**1**;

do i=is to &N;

if **0**<i-j<=&rkrg then s_Lower=s_Lower+DRG(i)*DIA(j); end;

end;

output;

end;

run;

/*Calculation of ASR*/

data ssa_c03(keep=TOTAL DIA_Gx_DRG DIA_Lx_DRG DRG1-DRG&N DIA1-DIA&N s_DRG s_DIA s_Upper s_Diago s_Lower R_c std_R_c expcsr null_e expest estimate expll expul ll ul);

merge ssa_c01 ssa_c02;

DIA_Lx_DRG=TOTAL-DIA_Gx_DRG;

/*Calculation of CSR*/

if DIA_Gx_DRG>=**0** & DIA_Lx_DRG>**0** then R_c=log(DIA_Gx_DRG/DIA_Lx_DRG); else R_c=**.**;

if DIA_Gx_DRG=**0** or DIA_Lx_DRG=**0** then std_R_c=**.**; else std_R_c=sqrt((**1**/DIA_Gx_DRG)+(**1**/DIA_Lx_DRG));

expcsr=exp(R_c);

/*Calculation of NSR*/

null_e=(s_Upper+s_Diago)/s_Lower;

if expcsr>=**0** & null_e>**0** then expest=expcsr/null_e;else expest=**.**;

estimate=log(expest);

ll=estimate-**1.96***std_R_c;

ul=estimate+**1.96***std_R_c;

expll=exp(ll);

expul=exp(ul);

run;

data out6(keep= csr nsr estimate sterr ll ul expest expll expul);

set ssa_c03;

csr=expcsr;

nsr=null_e;

sterr=std_R_c;

run;

proc append base=ssa_result data=out6;

run;

%END;

**%MEND** scm_simulation;

# **References**

1. Tsiropoulos I, Andersen M, Hallas J. Adverse events with use of antiepileptic drugs: a prescription and event symmetry analysis. Pharmacoepidemiol Drug Saf. 2009;18(6):483-491.

2. Hallas J. Evidence of depression provoked by cardiovascular medication: a prescription sequence symmetry analysis. Epidemiology. 1996;7(5):478-484.

3. Holt JD, Prentice RL. Survival Analyses in Twin Studies and Matched Pair Experiments. Biometrika. 1974;61(1):17-30.
